# Autoantibodies to BRAF, a new family of autoantibodies associated with rheumatoid arthritis

**DOI:** 10.1186/ar3165

**Published:** 2010-10-18

**Authors:** Caroline Charpin, Marielle Martin, Nathalie Balandraud, Jean Roudier, Isabelle Auger

**Affiliations:** 1 INSERM UMR 639, Parc scientifique et technologique de Luminy, 163 avenue de Luminy, 13288 Marseille cedex 09, France; 2 APHM La Conception, 264 rue Saint Pierre, 13385 Marseille, France

## Abstract

**Introduction:**

BRAF (v raf murine sarcoma viral oncogene homologue B1) is a serine-threonine kinase involved in the mitogen-activated protein kinase (MAPK) signalling pathway, known to be implicated in the production of pro-inflammatory cytokines.

We have observed that sera from rheumatoid arthritis (RA) patients recognize the BRAF's catalytic domain, which encompasses amino acids 416 to 766. Here, we identify peptide targets of anti-BRAF autoantibodies and test whether anti-BRAF autoantibodies may interfere with BRAF kinase activity.

**Methods:**

Anti-BRAF autoantibodies were detected by ELISA (enzyme-linked immunosorbent assay) in the serum of RA patients and controls, using 40 overlapping 20mer peptides encompassing the catalytic domain of BRAF as immunosorbents. To test whether autoantibodies to BRAF influence BRAF kinase activity, we developed an *in vitro *phosphorylation assay of MEK1 (mitogen extracellular regulated kinase), a major BRAF substrate. MEK1 phosphorylation by BRAF was tested in the presence of purified anti-BRAF autoantibodies from RA patients or control antibody.

**Results:**

We found that one BRAF peptide, P25 (656 to 675), is specifically recognized by autoantibodies from RA patients. Of interest, anti-P25 autoantibodies are detected in 21% of anti-CCP (cyclic citrullinated peptides) negative RA patients. Anti-BRAF autoantibodies activate the *in vitro *phosphorylation of MEK1 mediated by BRAF.

**Conclusions:**

Anti-BRAF autoantibodies from RA patients preferentially recognize one BRAF peptide: P25. Autoantibody responses to P25 are detected in 21% of anti-CCP negative RA patients. Most anti-BRAF autoantibodies activate BRAF kinase activity.

## Introduction

Rheumatoid arthritis (RA) is a chronic inflammatory joint disease with a prevalence of 0.5% worldwide [1]. The mechanisms leading to RA are unknown. The sera of RA patients contain many autoantibodies. The most characteristic are directed at citrullinated proteins (ACPA) [2]. ACPA recognize citrulline (a posttranslationally modified form of arginin) containing epitopes on various proteins, such as filaggrin, vimentin, and fibrinogen [3-6]. ACPAs can be detected by commercially available enzyme-linked immunoabsorbent assays using synthetic cyclic citrullinated peptides (CCP). Anti-CCP antibodies are detected in 60% of RA patients. Non-citrullinated proteins can also be the target of autoantibodies in RA [7,8].

By screening protein arrays, we found that BRAF (v raf murine sarcoma viral oncogene homologue B1) is a major non-itrullinated autoantigen recognized by 35% of RA patients' sera [8]. BRAF encodes a 766 amino acid serine-threonine kinase that contains a Raf-like Ras-binding domain (RBD encompassing amino acids 156 to 227), a protein kinase C-conserved region 1 domain (C1, amino acids 235 to 280) and a serine threonine protein kinase catalytic domain (amino acids 456 to 712) [9]. BRAF is involved in the mitogen-activated protein kinase (MAPK) signalling pathway, which regulates cell growth [10]. This pathway is also implicated in the production of proinflammatory cytokines leading to joint inflammation and destruction [11]. Activation of BRAF leads to activation of MEK1 and/or MEK2. These kinases are the major substrates of BRAF in mammalian cells [12].

We have observed that sera from RA patients recognize the BRAF's catalytic domain which encompasses amino acids 416 to 766. To identify peptide targets of anti-BRAF autoantibodies, we used 40 overlapping 20 mers encompassing the entire catalytic domain of BRAF to analyze RA sera. We found that one BRAF peptide, P25 (656 to 675), is specifically recognized by autoantibodies from RA patients. Of interest, anti-P25 autoantibodies are detected in 21% of anti-CCP negative RA patients.

To test whether autoantibodies to BRAF influence BRAF kinase activity, we developed a phosphorylation assay with BRAF, its substrate MEK1 and purified anti-BRAF autoantibodies from RA patients. We found that anti-BRAF autoantibodies activate the *in vitro *phosphorylation of MEK1 mediated by BRAF.

## Materials and methods

### RA patients

A total of 180 RA patients were chosen from the Rheumatology Ward at Hospital La Conception, Marseille, France. These patients fulfilled the 1987 American College of Rheumatology criteria for RA [13]. In every patient, HLA-DR genotyping and anti-CCP titration was obtained. One hundred, five RA patients were anti-CCP positive and 75 RA patients were anti-CCP negative. Ethical approval was obtained for this study; all participants gave their informed consent.

### Controls

Sixty-five patients with ankylosing spondylitis (AS) and 27 patients with psoriasis arthritis (PsA) from the Rheumatology Ward at Hospital La Conception, Marseille, 60 volunteers from the staffs of INSERM UMR 639 and the Marseille Blood Transfusion Center were tested. Ethical approval was obtained for this study; all participants gave their informed consent.

### Synthetic peptides

Forty 20-mer peptides, overlapping by 10 aminoacids and encompassing residues 416 to 766 of BRAF (locus NP_004324.1) were synthesized using the solid phase system and purified (Neosystem, Strasbourg, France). This segment from BRAF is polymorphic at position 599 where the usual valine residue can be replaced by a glutamate residue, a polymorphism associated with increased kinase activity and observed in human cancers [14,15].

**Figure 1:**
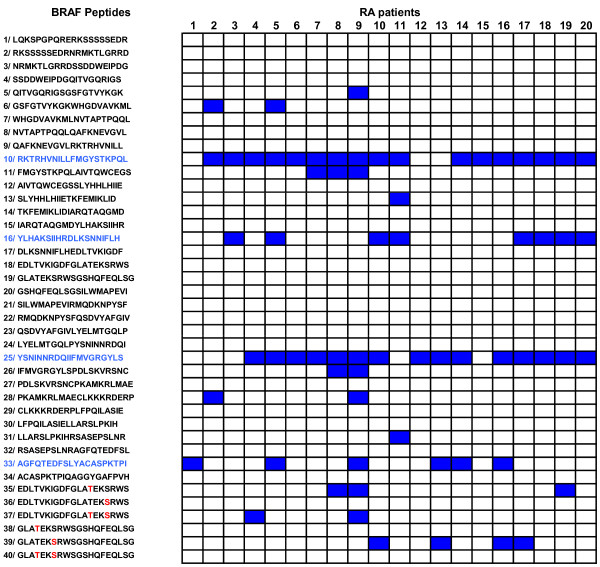
**Autoantibodies to BRAF recognize four linear peptides P10, P16, P25, P33**. Anti-BRAF autoantibodies from the sera of 20 RA patients were tested for binding to BRAF peptides by ELISA. After washing, peroxydase conjugated anti-human IgG was added. Optical density was read at 405 nm. Background OD was obtained by adding each serum to a well without peptide. Positive sera were defined by OD value higher than twice background OD (in blue).

Peptides P18 and P19 contain position 598 threonine and a position 601 serine residues which are the targets of phosphorylation during BRAF activation. Therefore, we synthesized both their native and phosphorylated forms, that is, P18 and its phosphorylated variants P35 (phosphorylated threonine 598), P36 (phosphorylated serine 601), P37 (both phosphorylated threonine 598 and serine 601), P19 and its phosphorylated variants P38 (phosphorylated threonine 598), P39 (phosphorylated serine 601) and P40 (both phosphorylated threonine 598 and serine 601). Phosphorylated residues are indicated in red in Figure 1.

### Detection of autoantibodies by ELISA

Plates were coated overnight with 10 μg peptide per well diluted in phosphate buffer saline (PBS), pH7.4. Plates were blocked with PBS containing 5% milk. Sera diluted to 1:100 in PBS were incubated for two hours on plates. After washing with 0.1% Tween 20, peroxydase conjugated anti-human IgG (Sigma Aldrich, ST Quentin Fallavier, France) was added. Optical density was read at 405 nm. Background OD was obtained by adding each serum to a well without protein. A positive serum was defined by an OD value more than twice background OD [7,8,16].

### Purification of autoantibodies to BRAF

Cyanogen bromide activated Sepharose 4B (Sigma Aldrich) was washed with 1 mM HCl and incubated with BRAF catalytic domain (amino acid 416-766, NP_004324, Invitrogen, Cergy Pontoise, France) in 0.1 M NaHCO3 and 0.5 M NaCl pH8 buffer overnight at 4°C. Free Sepharose groups were then blocked with 0.2 M Glycin pH8 for two hours at room temperature. Columns were washed at 4°C with the following buffers: 0.1 M NaHCO3, 0.5 M NaCl (pH8), then 0.5 M CH3COONa (pH4) buffer and finally with phosphate buffer pH7.

We selected 20 sera from RA patients, already known to contain autoantibodies to BRAF. Sera were incubated with 1 μg BRAF catalytic domain immobilized on sepharose. After washing, autoantibodies to BRAF were eluted in PBS pH2, neutralized in 1 M Tris and quantified.

### BRAF kinase assay

BRAF dependent phosphotransferase activity was measured in a kinase reaction using inactive recombinant MEK1 as a BRAF substrate (BRAF kinase assay Kit, chemiluminescence detection, Millipore Upstate Temecula, CA, USA). Briefly, MEK1 was incubated for 30 minutes at 30°C with 0.1 μg BRAF in working buffer in presence of 0.1 μg purified autoantibodies to BRAF.

For each patient, we included one positive phosphorylation control (incubation of inactive MEK1 with BRAF in working buffer) and one negative control (incubation of inactive MEK1 with BRAF in working buffer in presence of 0.1 μg control antibody C1). Control antibody C1 is an anti-human PAD4 autoantibody purified from RA patients' sera by the same technique as anti-BRAF.

Proteins were then separated on 8% SDS page and transferred onto PVDF membranes. Membranes were blocked, incubated with anti-phospho MEK1 antibody and revealed by chemiluminescence. Membranes were scanned with a Gene Flash (Syngene Europe, Cambridge, United Kingdom). Data were acquired with Gene Tools software (Syngene Europe, Cambridge, United Kingdom). Activation or inhibition of phosphorylation was evaluated by measuring the ratio test OD/positive control OD. A ratio of > 1 indicated activation while a ratio of < 1 indicated inhibition. Three kinase assays were performed separately for each patient and control.

### Statistical analysis

*P*-values were calculated using the Chi square Test. *P *< 0.01 was considered significant.

## Results

### Autoantibodies to BRAF recognize four linear epitopes on BRAF

To identify B cell epitopes on BRAF, we synthesized 40 overlapping 20 mer peptides encompassing the entire catalytic domain of BRAF. We screened these 40 peptides with the sera of 20 RA patients known to contain autoantibodies to BRAF. Among the 40 peptides, 14 were recognized by at least one of the tested sera. Four peptides, P10, P16, P25 and P33 were preferentially recognized by the sera of RA patients. Indeed, 17 sera recognized P10, 8 recognized P16, 15 recognized P25, and 6 recognized P33 (Figure 1).

### Peptide P25 on BRAF is specifically recognized by RA patients

To confirm these reactivities, we tested, using ELISA, the sera of 180 RA patients, 65 AS patients, 27 PsA patients and 60 healthy individuals on P10, P16, P25 and P33.

**Figure 2:**
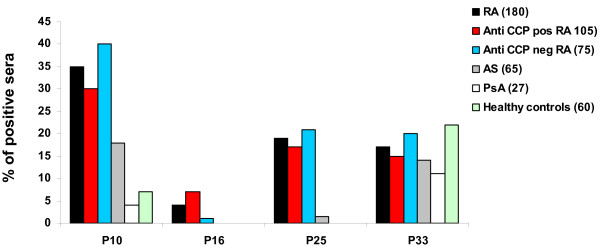
**Recognition of BRAF peptides in RA patients and controls**. Anti-BRAF autoantibodies from the sera of RA, AS, PsA patients and healthy controls were tested for binding to BRAF peptides by ELISA. After washing, peroxydase conjugated anti-human IgG was added. Optical density was read at 405 nm. Background OD was obtained by adding each serum to a well without peptide. Positive sera were defined by OD value higher than twice background OD.

Autoantibodies to p10 and p25 were found in RA patients more often than in controls (Figure 2). Autoantibodies to P10 were more sensitive but less specific for RA than P25. Indeed, 35% of RA patients' sera recognized P10 versus 18% of AS patients (*P *= 0.01), 4% of PsA patients (*P *= 0.001) and 7% of healthy individuals (*P *= 0.00002).

Autoantibodies to P25 were very specific for RA. Indeed, 19% of RA patients' sera recognized P25 versus 1.5% of AS patients (*P *= 0.0006), 0% of PsA patients (*P *= 0.01) and 0% of healthy individuals (*P *= 0.0003).

Autoantibodies to P16 were not commonly detected in RA. Indeed, only 4% of RA patients' sera recognized P16 versus 0% of AS patients, 0% of PsA patients and 0% of healthy individuals (*P *= 0.008 180 RA patients versus 152 controls). Finally, autoantibodies to P33 were less specific for RA. Indeed, 17% of RA patients, but also 14% of AS patients, 11% of PsA patients and 22% of healthy individuals recognized P33 (*P *= 0.85, 180 RA patients versus 152 controls).

### Peptide P25 on BRAF identifies RA in 21% of anti-CCP negative RA patients

Autoantibodies to P25 were analyzed by ELISA in 105 anti-CCP positive and 75 anti-CCP negative patients. Among anti-CCP positive patients, 17% recognized P25. Among anti-CCP negative patients, 21% recognized P25 (Figure 2).

### Autoantibodies to BRAF activate the phosphorylation of MEK1 by BRAF

To test whether autoantibodies directed to BRAF interfere with its enzymatic activity, we analyzed the phosphorylation of MEK1 in presence of BRAF and autoantibodies to BRAF purified from 20 RA patients.

Autoantibodies to BRAF were purified from RA patient's sera and their presence was confirmed by dot blot (data not shown). After quantification of purified autoantibodies to BRAF, we tested the phosphorylation of MEK1 in presence of BRAF and autoantibodies to BRAF purified from 20 RA patients. Phosphorylation of MEK1 by BRAF was detected by Western blotting and quantified. A mean ratio was obtained from three separate assays. A ratio of > 1 indicated activation, a ratio of < 1 indicated inhibition.

**Figure 3:**
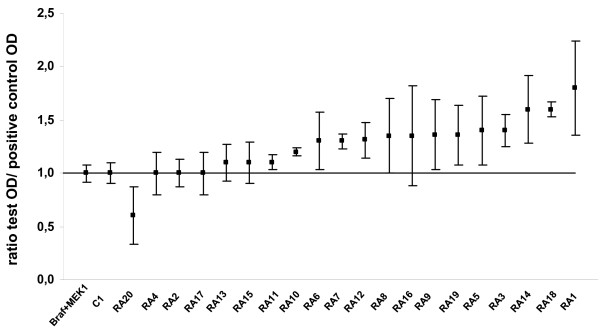
**Autoantibodies to BRAF activate the phosphorylation of MEK1**. Purified anti-BRAF autoantibodies from patients were incubated with BRAF and MEK1. For each patient, we included one positive control (BRAF and MEK1) and one negative control (BRAF and MEK1 and C1 control antibody). Activation or inhibition of MEK1 phosphorylation was detected by measuring the ratio test OD/positive control OD. Ratio > 1 indicated activation, ratio < 1 indicated inhibition. Bars showed the mean ratio ± SD.

Among 20 purified autoantibodies to BRAF, 1 inhibited and 13 activated MEK1 phosphorylation (Figure 3). We can't conclude for six autoantibodies to BRAF (RA4, RA2, RA17, RA13, RA15, and RA16). Indeed, a ratio of ≥1 was obtained but with a strong variability.

Among autoantibodies to BRAF that activated MEK1 phosphorylation, 10/13 were weak activators (1 < ratio < 1.5) and 3/13 were strong activators (ratio > 1.5).

No difference was observed in the recognition of BRAF peptides between autoantibodies to BRAF that inhibited or activated MEK1 phosphorylation (data not shown).

## Discussion

RA is an autoantibody mediated disease. The sera of patients with RA contain a family of highly disease-specific autoantibodies termed ACPA which recognize citrulline-centered peptidic epitopes on many proteins, like filaggrin, vimentin, and fibrin. Citrullyls are arginyl residues that have been converted from their native basic form into a neutral variant by a posttranslational modification called deimination, mediated by peptidylarginine deiminases. ACPAs can be detected by a commercial ELISA assay containing a synthetic cyclic citrullinated peptide and identify 60% of RA patients. However, non-citrullinated proteins can also be the target of autoantibodies in RA. For instance RA-associated HLA-DR alleles are associated with presence of autoantibodies to synovial calpastatin in RA patients' sera [7]. We also identified peptidyl arginine deiminase 4 (PAD4) as an RA specific autoantigen [8,16].

By using protein arrays, we recently found that BRAF is a major non-citrullinated autoantigen in RA. Indeed, 35% of RA patients' sera contain autoantibodies to BRAF's catalytic domain versus 4% of ankylosing spondylitis patients' and 6% of healthy individuals'. In this study, we mapped epitopes in the BRAF's catalytic domain and analysed the function of anti-BRAF autoantibodies.

To identify peptides targets of anti-BRAF autoantibodies, we used a direct ELISA using a set of synthetic peptides and the sera of RA patients. We identified two linear peptides on BRAF, P10 and P25, recognized preferentially by RA patients. Peptides P10 (506 to 525) and P25 (656 to 675) are located in the catalytic domain of BRAF.

P10 is more sensitive but less specific than P25. P10 is recognized by 35% of RA patients versus 11% of controls (*P *< 10^-7^, 180 RA patients versus 152 controls).

P25 might have diagnostic interest. Indeed, P25 is recognized by 19% of RA patients versus 0.7% of controls (*P *< 10^-7^, 180 RA patients versus 152 controls). Of higher interest, P25 is recognized by 21% of anti-CCP negative patients. Therefore, P25 could be used to diagnose RA in anti-CCP negative patients.

We then analysed the effects of anti-BRAF autoantibodies isolated from RA patients on the kinase activity of BRAF. Indeed, autoantibodies directed to an enzyme may interfere with its function. For example, we have recently shown that RA specific autoantibodies to PAD4 inhibit PAD4 mediated citrullination *in vitro *[16]. BRAF is also an interesting target for autoantibodies. Indeed, BRAF is a serine-threonine kinase involved in the transduction of mitogenic signals from the cell membrane to the nucleus. BRAF regulates the mitogen-activated protein kinase (MAPK) signalling cascade. A simplified and linear representation of this cascade comprises RAS, BRAF, MEK and ERK [17]. RAS activation is the first step in the activation of the MAPK cascade. Following RAS activation, BRAF is recruited to the cell membrane and then phosphorylates MEK in the cytoplasm. Activated MEK subsequently phosphorylates ERK, which translocates to the nucleus where it activates multiple transcription factors.

MAPKs are involved in signalling via the B cell antigen receptor, T cell receptor, Toll-like receptor and IL-1, IL-17 and TNFα receptors. MAPKs also play key roles in the production of pro-inflammatory cytokines (TNFα, IL-1, IL-6). In particular, p38 MAPK is expressed in the RA synovium and regulates the production of pro-inflammatory cytokines [18,19]. Inhibitors of p38 MAPK reduce osteoclast activation and prevent the development of collagen-induced arthritis in the mouse [20,21]. However, so far, p38 MAPK inhibition showed modest clinical efficacy in patients with RA [22].

To test whether autoantibodies to BRAF may influence BRAF activity as a kinase, we developed a phosphorylation assay using BRAF, MEK1 (its major substrate) and autoantibodies to BRAF purified from RA patients' sera. We observed that 65% of anti-BRAF autoantibodies activate phosphorylation of MEK1 by BRAF *in vitro*. Thus, we suggest that anti-BRAF autoantibodies could activate the MAP kinase pathway through BRAF, leading to pro-inflammatory cytokine production and joint inflammation.

**Figure 4:**
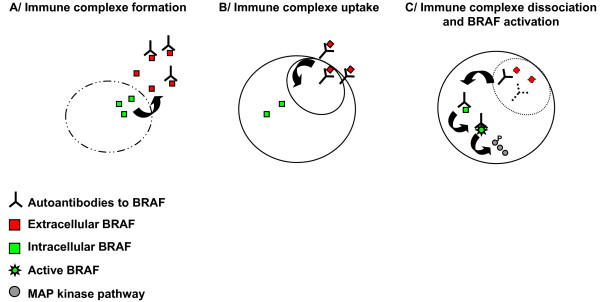
**A model to explain how autoantibodies to BRAF may activate BRAF**. **(A) **In RA, cell death may release BRAF outside the cell and BRAF could be recognized by autoantibodies. **(B) **Autoantibodies to BRAF might enter the cell as immune complexes. **(C) **Immune complexes could be dissociated. Autoantibodies to BRAF might access to intracellular BRAF, stabilize BRAF in active conformation by binding its catalytic domain and provoke MAP kinase activation.

We propose a model to explain how autoantibodies to BRAF may activate BRAF (Figure 4). Although BRAF is an intracellular protein, it could be recognized by autoantibodies outside the cell, because in RA, cell death may release BRAF from cells. Autoantibodies to BRAF might enter the cell later as immune complexes. After immune complexe dissociation, autoantibodies to BRAF become accessible to intracellular BRAF. BRAF activation is regulated by both its N and C terminal domains. The N terminal domain is responsible for the fixation of BRAF to RAS. The C terminal domain contains the substrate recognition sequence allowing MEK's phosphorylation [23,24]. The binding of autoantibodies to BRAF's catalytic domain may result in a change in BRAF conformation and stabilise BRAF in an active conformation allowing the kinase domain to contact its activators and substrates. Testing this model *in vivo *will be our next goal.

## Conclusions

We have described a new family of autoantibodies associated with RA. We have demonstrated here that these autoantibodies activate BRAF, which is the first step in MAP kinase activation. Fine epitope mapping on BRAF enabled us to identify one peptide epitope, P25 which may prove interesting in the diagnostic of RA, especially in anti-CCP negative patients.

## Abbreviations

ACPA: anti-citrullinated peptide antibodies; AS: ankylosing spondylitis; BRAF: v raf murine sarcoma viral oncogene homologue B1; CCP: cyclic citrullinated peptides; MAPKs: mitogen-activated protein kinases; MEK1: mitogen extracellular regulated kinase; PAD4: peptidyl arginine deiminase 4; PBS: phosphate buffer saline; PsA: psoriasis arthritis; RA: rheumatoid arthritis; SD: standard deviation.

## Competing interests

The authors declare that they have no competing interests. A patent was submitted in March 2010. Submission number: 788471. PCT application number PCT/EP2010/054087. Receiving Office European Patent Office, The Hague: BCT100057BA.

## Authors' contributions

All authors were involved in drafting the article, and revising it critically for important intellectual content. IA and JR contributed to study conception and design. IA, CC, MM and NB contributed to acquisition of data. IA, CC and JR contributed to analysis and interpretation of data. All authors read and approved the final manuscript.
